# Rituximab in stiff-person syndrome with glutamic acid decarboxylase 65 autoantibody: a systematic review

**DOI:** 10.1007/s00415-025-13157-2

**Published:** 2025-05-24

**Authors:** Antonia Pignolo, Claudia Vinciguerra, Roberto Monastero, Nicasio Rini, Angelo Torrente, Carmela Rita Balistreri, Filippo Brighina, Vincenzo Di Stefano

**Affiliations:** 1 Department of Biomedicine, Neuroscience, and Advanced Diagnostics (Bi.N.D.), University of Palermo, 90129 Palermo, Italy; 2 Neurology Unit, Department of Medicine, Surgery and Dentistry “Scuola Medica Salernitana”, University Hospital San Giovanni Di Dio E Ruggi D’Aragona, 84131 Salerno, Italy

**Keywords:** Rituximab, Stiff-person syndrome, Anti-GAD antibody, Glutamic acid decarboxylase

## Abstract

**Background:**

Stiff-person syndrome (SPS) is a rare autoimmune neurological disorder characterized by muscle rigidity and painful spasms, predominantly affecting young women. It is often associated with high titers of anti-glutamic acid decarboxylase (GAD) 65 antibodies. Current treatments for SPS include symptomatic therapies and immunomodulatory approaches, but there is a need for more effective therapies because many patients show incomplete responses and disease progression.

**Methods:**

The systematic review followed the Preferred Reporting Items for Systematic Reviews and Meta-Analyses (PRISMA) guidelines, with a literature search of PubMed, Web of Knowledge, Google Scholar, and Science Direct. Studies evaluating efficacy, safety, dosage, and impact on concomitant treatments of Rituximab (RTX) in SPS were selected. Data on anti-GAD titers were also analyzed.

**Results:**

Fourteen studies published between July 2005 and October 2022 were selected. The studies included 30 SPS patients treated with RTX. Data were heterogeneous regarding dosage, administration schedule, and patient assessment. RTX was generally well-tolerated, with rare side effects, including infusion reactions or infections. Significant clinical improvement occurred in most patients, with a small proportion achieving complete remission. Anti-GAD antibody titers decreased in some studies, with no consistent correlation with clinical outcomes.

**Conclusions:**

Evidence supporting the efficacy of RTX in SPS is limited by the small sample sizes of the included studies and the variability in treatment protocols. However, RTX has shown efficacy for clinical improvement. Correlation with anti-GAD titers remains still unclear. Further randomized controlled trials are needed to confirm RTX as an established treatment for SPS.

**Supplementary Information:**

The online version contains supplementary material available at 10.1007/s00415-025-13157-2.

## Introduction

Stiff-person syndrome (SPS) is a rare and chronic neurological disease that primarily affects women, usually manifesting between the third and sixth decades of life, most commonly above the age of 20 years [1, 2]. Clinically, SPS is characterized by episodic or persistent muscle stiffness and painful spasms, predominantly involving the trunk and proximal limbs. These symptoms may fluctuate over time and can be exacerbated by emotional stress or external stimuli such as noise or tactile contact [3, 4]. Over time, rigidity and spasms may impair gait, increase the risk of falls, and affect daily activities such as turning in bed or breathing, sometimes leading to respiratory compromise and the need for intensive care [1, 4]. Ocular manifestations such as nystagmus, slow saccades, and impaired initiation of eye movements—sometimes referred to as “stiff eyes”—have also been reported in a subset of patients [5]. SPS belongs to the “glutamic acid decarboxylase antibody-spectrum disorders” (GAD-SD), a group of autoimmune disorders that includes various neurological conditions, such as cerebellar ataxia, limbic encephalitis, autoimmune epilepsy, myoclonus, and nystagmus, as well as systemic autoimmune diseases such as type 1 diabetes mellitus (T1DM), thyroiditis, psoriasis, and myasthenia gravis (MG) [3]. These disorders are immunologically linked through the presence of circulating autoantibodies targeting proteins essential for inhibitory synaptic transmission, including glutamic acid decarboxylase (GAD), glycine receptor α-subunit (GLRA1), GABA_A_ receptor-associated protein (GABAAR), and amphiphysin [2–4, 6, 7].

The pathophysiological mechanism of SPS is not fully understood. It is predominantly autoimmune, involving the dysfunction of GABAergic neurotransmission within the central nervous system (CNS) [3, 8–10]. GAD, the enzyme that catalyzes the conversion of glutamate into the inhibitory neurotransmitter GABA, plays a central role in this process [3, 6–8]. Moreover, there are two isoforms of GAD, GAD65 and GAD67. GAD65 is predominantly concentrated in nerve terminals and regulates the activity-dependent GABA synthesis when postsynaptic inhibition is required. Meanwhile, GAD67 maintains cytosolic levels of GABA in neurons [11, 12]. In addition, GAD is an intracellular enzyme widely expressed in the CNS whose physiological function is the decarboxylation of glutamate to GABA, the main inhibitory neurotransmitter [8]. High titers of GAD65 antibodies are frequently associated with type 1 diabetes and a broad spectrum of autoimmune neurological disorders [9].

The detection of anti-GAD antibodies in serum or cerebrospinal fluid (CSF) is a supporting criterion for SPS diagnosis, but seronegative SPS has been reported [3]. Indeed, although a high titer of anti-GAD65 antibodies supports the diagnosis, low titers may lead to false positives and even misdiagnosis [1]. Furthermore, the technique used for anti-GAD Ab detection significantly influences the results: a high titer is defined as > 10,000 IU/ml detected by enzyme-linked immunosorbent assay (ELISA) or > 20 nmol/l by radioimmunoassay (RIA) in serum; also, the presence of anti-GAD antibodies in the CSF is confirmatory [1].

Due to the partial and often inadequate response to symptomatic treatments such as benzodiazepines (e.g., clonazepam), baclofen, tizanidine, piracetam, and botulinum toxin [13], immunotherapy has emerged as a central strategy in SPS management. First-line immunomodulatory options include corticosteroids, intravenous immunoglobulin (IVIg), and plasma exchange (PE) [14]. Although IVIg has shown clinical benefit in several studies, its long-term efficacy remains under investigation [15]. In selected refractory cases, subcutaneous immunoglobulins (SCIg) or autologous hematopoietic stem cell transplantation (AHSCT) have been attempted [16, 17]. PE is typically reserved for patients unresponsive to IVIg, with clinical improvement reported in up to 56% of cases [18]. Additionally, new therapeutic options such as efgartigimod—used in SPS patients with concurrent MG—have demonstrated promising results, with rapid clinical improvement and no severe adverse events reported [19].

Despite these therapeutic strategies, many patients continue to experience debilitating symptoms and require long-term immunotherapy. Given the central role of autoreactive B cells in autoimmune pathogenesis, therapies targeting B cells have become increasingly attractive. Rituximab (RTX), a chimeric monoclonal antibody directed against the CD20 antigen expressed on pre-B and mature B lymphocytes, promotes B-cell depletion through apoptosis, antibody-dependent cell-mediated cytotoxicity (ADCC), and complement-mediated cytolysis. Importantly, RTX does not interfere with immunoglobulin production by plasma cells or B-cell regeneration from progenitor cells [20, 21]. Although some case reports and small studies suggest that RTX may benefit patients with SPS, dosing schedules are not standardized and clinical outcomes remain variable, with some cases reporting significant adverse events [14].

This systematic review aims to evaluate the efficacy and safety of rituximab in patients with SPS, with particular attention to its clinical impact, duration of effect, adverse reactions, and potential to reduce reliance on other concurrent treatments.

## Methods

The included studies were identified by searching all relevant medical articles in English through the following electronic databases: Web of Knowledge, PubMed, Google Scholar, and ScienceDirect (until January 2024). The specific search strategy combined the terms “stiff-person syndrome”, “stiff person syndrome” and “rituximab” but also “stiff-man syndrome”, “stiff man syndrome” and “rituximab”. All articles related to the use of RTX in SPS with seropositivity to GAD65 antibodies (Ab) involving human subjects, including case reports, were collected. Review articles and editorials were also considered to search for any additional bibliographic entries. Studies involving nonhuman samples were excluded. The review was conducted using the PRISMA reporting guidelines.

Two investigators (AP, AT) first reviewed the titles and abstracts of all retrieved articles for duplication and then examined all potentially relevant articles independently to determine the final selection of studies. In case of discrepancies, these were resolved by consensus of all authors. Each selected article was assessed for its evidence level using the PICO tool strategy (patient/problem, intervention, comparison, outcome). Data were collected in the following areas: study characteristics, follow-up method, baseline demographic data, and symptom prevalence. The following data were collected and recorded: study design (case reports, retrospective study, double-blind crossover study), sample size, RTX dosage, clinical outcome of the study, efficacy, effects on antibodies titers, and study limitations.

## Results

**Fig. 1:**
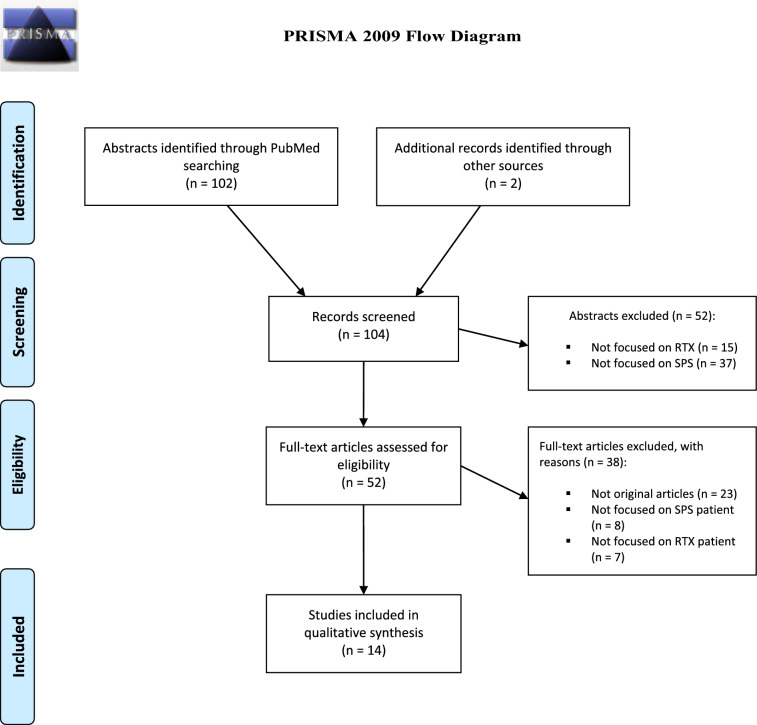
PRISMA flow diagram. The database search yielded 104 articles, of which 52 were excluded after reading the abstracts and 52 were eligible for inclusion in the systematic review. Of these, 38 were excluded after reading the full article, 23 because they were not original articles, 8 not focused on SPS, and 7 not focused on RTX. Finally, 14 studies met the established inclusion criteria, being 11 case reports, 1 retrospective observational study, and 2 double-blind, placebo-controlled studies

The database search yielded 104 articles, of which 52 were excluded after reading the abstracts, and 52 were eligible for inclusion in the systematic review. Of these, 38 were excluded after reading the full article, 3 because they were not original articles, 8 not focused on SPS, and 7 not focused on RTX. Finally, 14 met the established inclusion criteria (see the flowchart for the systematic review of electronic databases in Fig. 1). The 14 included studies were published in a relatively short period (i.e., July 2005–October 2022). For each selected article concerning RTX treatment, data collected included the number and regimen of infusions used for RTX therapy, antibody titers before and after treatment, safety, and SPS status post-intervention. The quality of data and outcome measures varied among the included studies, with heterogeneity in outcome measures, assessment of anti-GAD Ab titers, treatment schedule, and dosages. To date, there are no open-label prospective studies on the use of RTX in SPS, and the available evidence consists of a small number of patients collected from, for the most part, case reports, and some double-blind, placebo-controlled study and retrospective studies. Supplementary Table 1 shows details regarding all the included studies results.

### Included studies design

Among the 14 selected studies, 11 were case reports [2, 5, 22–30], 1 retrospective observational study [11], and 2 were double-blind, placebo-controlled studies [31, 32].

### Number of patients, RTX dose, and frequency

Thirty SPS patients from 14 studies were treated with RTX. Dosage and administration were different among the studies. Considering the global number of patients included in this review, the most common induction regimen was 1000 mg given twice with a 2-week interval (total of 2000 mg) used in 19 patients [22, 26, 28, 30–32], followed by 375 mg/m2 weekly for four weeks (total 1.5 g) or twice with a 2-week interval in 5 patients [5, 23–25, 27]. A few studies have used lower doses of RTX (375 mg/m2 about once time in a month in 1 patient [2] or 500 mg/m2, twice with a 2-week interval in other 1 [29]. In one study, the exact dosage of RTX administered to 4 patients involved in the study is unspecified [11]. Moreover, we have not considered the studies including patients who received RTX as add-on therapy with different other immunotherapies (e.g., glucocorticoids and/or IVIg, mycophenolate mofetil). However, the details on RTX dosing in induction, relapse management, and maintenance were often unclear in some studies [11, 27, 29]. Sevy et al. specified that maintenance RTX administration, after 6 months, is necessary to avoid clinical relapses [30].

### Efficacy and clinical outcome

**Fig. 2:**
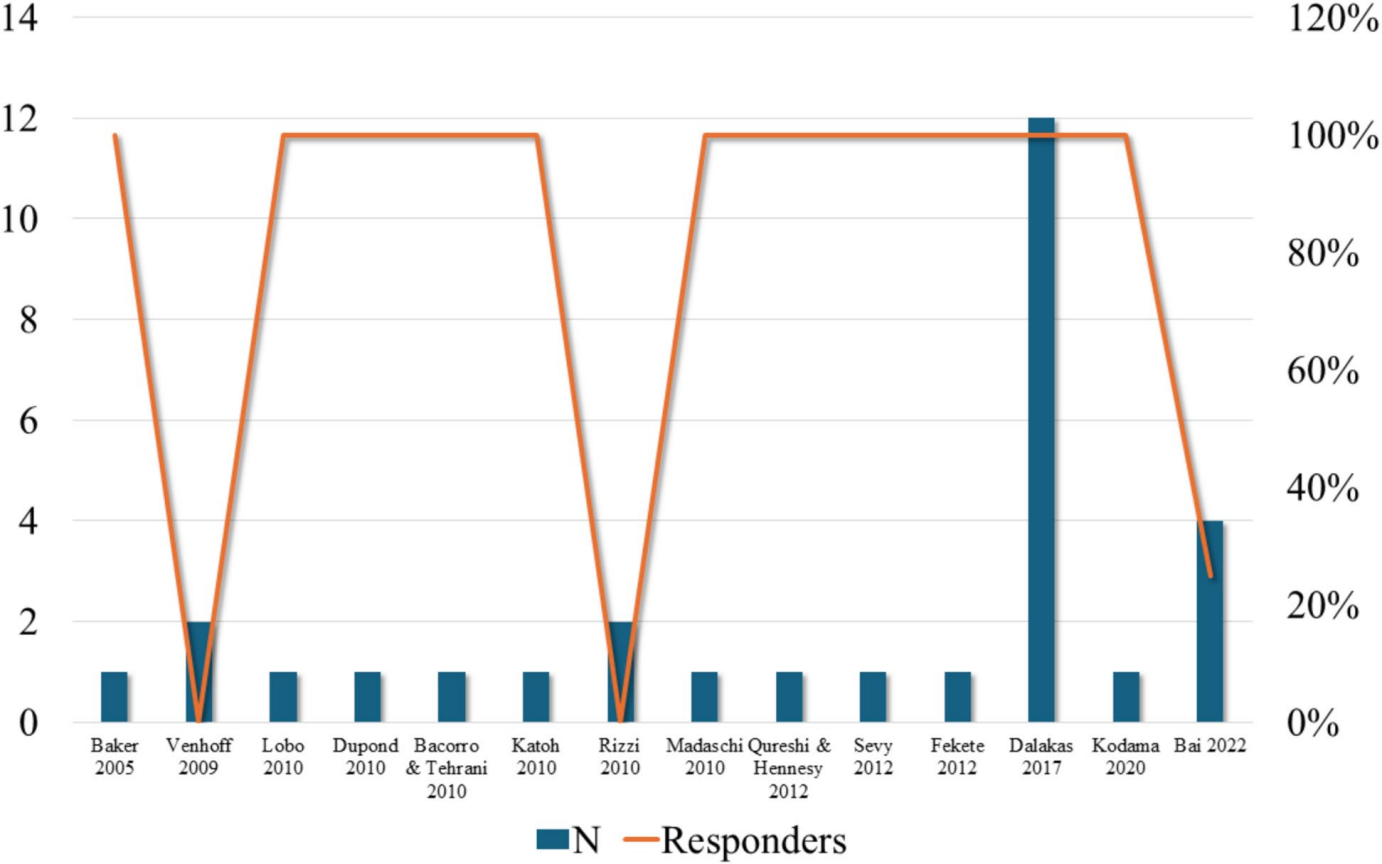
Representation of the number of patients included (N, left y-axis) in each study (x-axis) and the clinical responders’ rate (%, right y-axis) to the treatment with RTX

Overall, significant clinical improvement was reported in 77% of patients (23/30), the response rate of the different studies is shown in Table 1 and Fig. 2. The time of last follow-up to evaluate the clinical response was variable, with a median of 9 months and an interquartile range of 6–12 months. Complete remission of SPS was reported in 3 out of 23 patients (13%). The outcome measures were heterogeneous among the included studies, but RTX resulted in a sustained clinical improvement (i.e., an improvement on the modified Rankin Scale [mRS]) and reduction or cessation of benzodiazepines and other antispasmodic and immunologic therapies. We must specify the extreme variability of the scales used as outcome measures and for the clinical assessment among the studies. Some studies used scales to quantify stiffness and spasms, as the stiffness index and spasms index [30, 31], while others used more specific ones, for example, Madaschi et al. evaluated specifically ocular motility [5, 28]. In some studies, authors used generical and multidimensional scale as the “Modified Ranking Scale” (mRS) [11] or “Scale for Short Form Health Survey 36"(SF-36) [32] to show improvement; in others the authors did not specify the clinical scale used [33]. As shown in the supplementary Table 1, in a retrospective observational study, only 4 patients among 55 received RTX in monotherapy, obtaining a doubtful, not really predictable clinical improvement. One of 4 patients reported a clinical improvement (25% responders) [11]. In a double-blind, placebo-controlled study 6 months after RTX treatment, the stiffness index was equally reduced in both groups. QOL scores improved significantly at 3 months in both groups, but not at 6 months [31]. In another double-blind, randomized, placebo-controlled crossover study was not shown a substantial clinical improvement after RTX in the patients (0% responders) [32]. Considering all the case reports included in this review, some studies demonstrated the clinical improvement with resolution of stiffness after RTX infusion; in contrast, other studies did not achieve a sustained reduction in stiffness, spasms, simultaneous painful contractions of agonist and antagonist muscles, and clonus [2, 5, 22–30] obtaining a global response rate of 83%.

**Table 1:** Summary of the number of patients included (N) in each study and the clinical responders’ rate (%) to the treatment with RTX. The last column shows the last follow-up at which the clinical response was evaluated; for some studies the time was not specified (NS)

Study	*N*	Responders	Last follow-up (months)
Baker 2005	1	100%	3
Venhoff 2009	2	0%	13
Lobo 2010	1	100%	12
Dupond 2010	1	100%	NS
Bacorro and Tehrani 2010	1	100%	6
Katoh 2010	1	100%	3
Rizzi 2010	2	0%	12
Madaschi 2010	1	100%	12
Qureshi and Hennesy 2012	1	100%	12
Sevy 2012	1	100%	6
Fekete 2012	1	100%	NS
Dalakas 2017	12	100%	6
Kodama 2020	1	100%	NS
Bai 2022	4	25%	NS

### Side effects

RTX was generally well-tolerated. Previous studies indicated its safety, focusing on the frequency of study-related adverse events in SPS patients [9, 14, 34, 35]. The most common side effects included infusion reactions and infections due to immunosuppression and neutropenia, such as respiratory tract infections, breathing problems, and arrhythmia. No serious adverse events were reported in the selected studies, except for a mild Pneumocystis Jiroveci lung infection, promptly treated with antibiotics [27].

### Glutamic acid decarboxylase (GAD) antibody titers

Anti-GAD antibody titers have been monitored in most studies [22–30]. From the available data, it is unclear whether treatment with RTX leads to a subsequent reduction of anti-GAD titers. Three studies have shown a decrease in anti-GAD antibody titers after RTX [23, 27, 31], but in others the levels remained variable and did not significantly decrease [22, 25, 26, 28, 29] or showed a paradoxical increase in anti-GAD titer [24, 30]. Dupond et al. also detected anti-amphiphysin concentration, which was undetectable after RTX treatment, reducing from 1/6400 IU/ml to 1/1600 IU/ml, while anti-GAD concentration was high, with a titer ranging between 70.74 IU/mL and 142.7 IU/mL [25]. In addition, some studies have not reported anti-GAD or other Ab titers modification after RTX treatment [2, 11, 32]. Table 2 summarizes the main results obtained by the literature review.

**Table 2:** Summary of the main results of the included studies

Study design	RTX dosage	Side effects	Clinical outcome	Response rate	RTX effects on Ab titers	Main limitations
11 case reports; 1 retrospective observational study; 2 double-blind, placebo-controlled studies	From 375 to 500 mg/m^2^ body surface area given two to four times every week or every two weeks to 1 g given twice every two weeks	Most studies did not report side effects, 1 patient reported pneumonia, 1 patient referred minor side effects (fever, chills, nausea, pruritus, mild hypotension, and headache)	Various clinical benefits, with stiffness reduction, gait improvement, or reduction in the use of BDZ. A substantial minority of patients did not obtain clinical improvements, 23% of patients (7/30)	Clinical improvement was reported in 77% of patients (23/30). Complete remission of SPS was reported in 3 out of 23 patients (13%)	Various response, from CSF and plasma titers reduction to serum levels stability or even increase	Low number of patients; extreme variability of outcomes; missing report of RTX dosages, clinical assessment scales used, ab titers

*BDZ* benzodiazepines, *CSF* cerebrospinal fluid, *GAD* glutamic acid decarboxylase antibody, *RTX* rituximab, *Ab* antibodies

## Discussion

SPS is a rare and slowly progressive autoimmune neurological disorder characterized by central and peripheral neuronal hyperexcitability, primarily due to impaired GABAergic neurotransmission and high titers of anti-GAD antibodies [36]. Standard treatments include symptomatic drugs such as antispasmodics, botulinum toxin injections, and benzodiazepines [14]. IVIg, PE, immunosuppressants, and FcRn inhibitors have been employed in SPS, although IVIg, administered as a chronic therapy, has shown limited efficacy [3, 19, 37, 38].

Data from this systematic review highlighted how RTX, a monoclonal antibody targeting CD20 on B cells, has shown potential to improve clinical outcomes for SPS patients. However, a wide and significant variability has been found in terms of dosage, treatment schedule, and patient assessment. RTX is generally considered safe, although serious adverse events, albeit rare, can occur, often related to the infusion process and usually mitigated by premedication. Although concerns about prolonged immunosuppression remain, severe side effects are uncommon. The efficacy of RTX in GAD-related disorders presents inconsistent results, with only two small randomized controlled trials [31, 32] reporting partial clinical improvements. One retrospective observational study suggested that most patients showed clinical improvement after immunotherapy, although complete recovery was observed in only a few cases; this study also included other immunotherapies such as glucocorticoids, IVIg, and mycophenolate mofetil [11].

Other available research, [2, 22–30, 39] indicates that RTX often leads to sustained clinical improvement, longer intervals between relapses, and a reduction in other therapies. Nevertheless, the optimal RTX dosage for management of SPS exacerbations, induction, and maintenance therapy has not been validated and needs further randomized trials. The variability in results may result from the heterogeneity of anti-GAD disorders, suggesting that RTX may benefit only specific subgroups of patients, with factors such as the subjective nature of symptoms, frequent fluctuations, behavioral symptoms, and different pathogenic mechanisms contributing to varying treatment responses in SPS. Whether RTX induces serologic remission in refractory SPS remains unclear.

Moreover, some studies reported a decrease in anti-GAD antibody levels following RTX treatment, although this reduction does not consistently correlate with clinical outcomes. For instance, Dakalas et al. (2017) observed a significant reduction in anti-GAD antibody titers with RTX, even if the therapy was not more effective than placebo; thus, the reduction in antibodies may not directly reflect the therapeutic efficacy of RTX [31]. In contrast, while some studies have observed a decrease in anti-GAD antibodies after treatment, others have reported persistent elevation or even paradoxical increases despite clinical improvement [24, 30], raising the possibility that anti-GAD antibody titers are not directly related to clinical presentation but to the disease duration. This review is focused on SPS patients with positivity of anti-GAD antibodies. Despite this, in literature, there is some limited evidence of using RTX treatment observed in seronegative SPS patient. For example, as demonstrated by Horiuchi et al., RTX induced a clinical improvement (i.e. muscle spasms, myoclonus, and weakness reduction) in a seronegative SPS patient with Waldenström macroglobulinemia, without side effects, particularly no worsening of macroglobulinemia [40]. Dupond et al. demonstrated the efficacy of RTX in a patient with multiple autoimmune conditions, including SPS, in which RTX led to remission and suppression of anti-amphiphysin antibodies but not anti-GAD [25]. The latter result supports the efficacy of RTX in more severe forms of SPS, especially when associated with additional autoimmune conditions. Therefore, despite no clear evidence was found for the early initiation of RTX, it could be considered as an effective immunotherapy for patients with severe disease forms or in cases of overlapping of different GAD-associated disorders.

### Limitations

Most of the analyzed studies in this systematic review have several limitations that may hinder the evaluation of RTX efficacy in SPS. First, there is considerable heterogeneity in outcome measures and the extent of disease involvement among studies. Although clinical instruments such as the Stiffness Index and Heightened Sensitivity Scale are available, these scales may still be insensitive. Furthermore, apart from the recently proposed SPS-ADL [19], there are no validated tools to assess the impact of SPS on daily life. Another limitation stems from the variability in treatment approaches among patients receiving RTX; for ethical reasons, many patients underwent concomitant treatments, such as IVIg infusions or PE, during the studies, which could affect the interpretation of results. In addition, the number of RTX cycles and administration schedules varied widely among studies. Consequently, the management of SPS remains empirical and based on individual experiences, highlighting the need for a definitive standardized therapeutic approach.

## Conclusions, remarks, and future directions

RTX represents a promising therapeutic option for SPS, particularly in patients who do not respond to conventional treatments. Although RTX has shown potential in reducing disease activity and improving mobility, its effects on other clinical aspects, such as rigidity, pain, and overall quality of life, remain unclear. The variability in clinical outcomes, likely due to the various manifestations and underlying mechanisms of SPS, suggests that RTX may benefit only specific subgroups of patients. The lack of a consistent correlation between anti-GAD65 antibody titers and clinical improvement further complicates the assessment of RTX efficacy. Current research on RTX in SPS is limited by small sample sizes, heterogeneity of study design, and variability in outcome measures. The inconsistent relationship between antibody levels and clinical outcomes indicates the need for more precise biomarkers to assess treatment effectiveness. However, RTX does not seem to be sufficient for maintaining remission because the patient still needs to use benzodiazepines as symptomatic drugs. In addition, the absence of standardized treatment protocols makes it difficult to draw firm conclusions about the optimal use of RTX. Finally, no consistent data have been reported on the use of RTX in seronegative individuals. Therefore, the evidence for efficacy in this subgroup remains weak, largely due to low internal validity and small sample sizes in most studies.

Future research should prioritize larger, randomized controlled trials with standardized protocols to determine the optimal dosing, timing, and patient subgroups that most likely benefit from RTX. Addressing these limitations is crucial for strengthening the evidence base and guiding clinical practice. In summary, while RTX offers hope for improving the management of SPS, more rigorous studies are needed to establish its definitive role in this complex and challenging disorder.

## Supplementary Information

Below is the link to the electronic supplementary material.

Supplementary file1 (DOCX 29 KB)
