# Supplementary material for: Rituximab in stiff-person syndrome with glutamic acid decarboxylase 65 autoantibody: a systematic review
Source: J Neurol. 2025 May 24;272(6):417. doi: 10.1007/s00415-025-13157-2 (PMC12103359; doi:10.1007/s00415-025-13157-2)
Supplement: Supplementary file 1 — Supplementary file1 (DOCX 29 KB) [file 415_2025_13157_MOESM1_ESM.docx]

**Supplementary table 1 –** Selected studies on the use of RTX in SPS applying the PICO procedure.

| ***Study*** | ***Study Design*** | ***Sample size*** | ***Patient’s characteristics*** | ***RTX treatment or dosage*** | ***Side effect*** | ***Clinical outcome*** | ***Responder rate*** | ***RTX effects on antibodies titers*** | ***Follow-up*** | ***Limitations*** |
| --- | --- | --- | --- | --- | --- | --- | --- | --- | --- | --- |
| **Baker 2005** | Case report | 1 | 41-year-old female patient with 3-year disease history; relapse after 3 months | RTX 375 mg/m^2^ body surface area given four times with an interval of 1 week; after 3 months, received RTX 375 mg/m^2^ | Not reported side effects | Clinical improvement with resolution of stiffness 15 days after the infusion | 100% | Anti-GAD ab CSF levels were undetectable 17 days after the first RTX infusion (from 3,2 IU /ml to 0) and serum anti-GAD levels remained low (between 0,1 and 0,3 IU/ml) | 15 days after the infusion the patient resolved stiffness. Symptoms reappear after the sixth week, so she received a four-week course of RXT in the same dosage at weekly intervals, with a clinical improvement after 14 days. | Low number of patients. Extreme variability of outcome measures. |
| **Venhoff 2009** | Double-blind, randomized, placebo controlled crossover study | 2 | 34-year-old male monozygotic twins patients, with 5-year disease history; comorbidities: thyroiditis | RTX 1 gr given twice with an interval of 2 weeks vs placebo, repeated after 54 weeks | No side effects | Support a genetic factor in the etiology of SPS but not a clinical benefit. They used Short Form Health Survey 36" (SF-36) without showing a substantial clincal improvement after RTX | 0% | Anti–GAD ab titers are not particularly affected by the treatment (from 205 IU /ml to 103 IU/ml) | A prescheduled switch between RTX and placebo arms was at week 18 and prescheduled follow-up visits at weeks 36 and 54. At week 54, both twins received an open-label second course of RTX 2 weeks apart. | Low number of patients.  Extreme variability of outcome measures and clinical assessment scales, together with missing ab titers. |
| **Lobo 2010** | Case report | 1 | 41-year-old female patient, with 7-year disease history | RTX 375 mg/m^2^ body surface area given twice with an interval of 2 weeks | No side effects | Clinical improvement with decreased muscular tonus requiring smaller dose of intravenous BDZ | 100% | Anti-GAD  serum  ab increased after treatment from 35 to 149 IU/mL after first infusion and 198 IU/mL after the second infusion | 2 days after the infusion of RTX she started showing a decrease in the muscular tonus and required a progressively smaller dose of intravenous BDZ. The effects of the treatment lasted for about 1 year. | Low number of patients.  Extreme variability of outcome measures and clinical assessment scales. |
| **Dupond 2010** | Case report | 1 | 50-year-old male patient; comorbidities: thymoma, DM, thyroiditis; relapse after 8 months | RTX 375 mg/ m^2^ given four times with an interval of 1 week. After 11 months, received a single dose RTX 375 mg/ m^2^ | No side effects | Clinical improvement with spasms suppression but with residual stiffness affecting the back, the abdomen and limbs | 100% | Serum anti-amphiphysin  Ab reduced from 1/6400 IU/ml to 1/1600 IU/ml. Anti-GAD antibodies remained strongly positive (from 90 IU /ml to140 IU/ml) | Following treatment with RTX the patient achieved a complete remission | Low number of patients.  Extreme variability of outcome measures and clinical assessment scales. |
| **Bacorro and Tehrani 2010** | Case report | 1 | 62-year-old male patient with 1-year disease history; comorbidities: DM and hypertension | RTX 1 gr given twice with an interval of 1 week | No side effects | Clinical improvement | 100% | Anti-GAD ab levels remained elevated, from 102,86 IU/mL to 89,17 IU/mL after 3 months | Clinical improvement was significant after 2 doses of RTX and continued 6 months after, despite persistence of the anti-GAD titer | Low number of patients.  Extreme variability of outcome measures and clinical assessment scales. |
| **Katoh 2010** | Case report | 1 | 66-year-old female patient with 7-year disease history; comorbidities: DM and TAO | RTX 375 mg/ m^2^ given twice with interval of 1 month and repeated 1 dose after 3 months | Mild pneumonia due to Pneumocystis jiroveci infection, which immediately recovered in response to sulfamethoxazole/trimethoprim | Improvement of muscle spasms due to SPS and ophthalmoplegia | 100% | Anti-GAD ab profoundly slowly  decreased after 6 weeks after treatment (from 19,9 IU/ml to < 1,5 IU/mL) | The efficacy did not continue for longer than 6 weeks. RTX was repeated after 3 months because of slight increase in the frequency of muscle spasms | Low number of patients.  Extreme variability of outcome measures and clinical assessment scales. Missing RTX dosages and ab titers |
| **Rizzi 2010** | Case report | 2 | 2 monozygotic twins patients with 7-year and 5-year disease history respectively | RTX 1 gr given twice with an interval of 2 weeks | No side effects | The clinical course in both patients remained unchanged | 0% | A 2-fold decline of anti-GAD titer from 1/256,000 to 1/128,000 was observed in both patients 8 weeks after RTX and persisted up to week 36 | During 1 year of follow-up clinical improvements were not observed | Low number of patients.  Extreme variability of outcome measures and clinical assessment scales. Missing ab titers. |
| **Madaschi 2010** | Case report | 1 | 62-year-old male patient with 4-year disease history; comorbidities: Graves-Basedow disease and TAO | RTX 1 gr given twice with an interval of 2 weeks | Fever, chills, nausea, pruritus, hypotension, and headache. No serious side effects | During the subsequent 4 months TAO was resolved with a sustained improvement of the spastic paraparesis | 100% | Anti–GAD ab titers remained high (from 4,300 to 3,600 arbitrary units) | The spastic paraparesis with SPS showed a pronounced and sustained improvement for 1 year after RTX | Low number of patients.  Extreme variability of outcome measures and clinical assessment scales. Missing ab titers. |
| **Qureshi and Hennesy 2012** | Case report | 1 | 56-year- old male patient with 6-year disease history; comorbidities: hyperthyroidism, DM and hypertension | RTX 375 mg/ m^2^ 4 doses over 3 months | Not reported side effects | Clinical improvement with decrease in limb and abdominal muscle stiffness and improvement of dysarthria | 100% | Anti-GAD ab titers reduced from >1,000 to 400 IU/ml 1 year after rtx | Clinical improvement lasted 1 year after RTX | Low number of patients.  Extreme variability of outcome measures and clinical assessment scales. Missing RTX dosage. |
| **Sevy 2012** | Case report | 1 | 50-year-old female patient; with 5-year disease history; comorbidities:DM1, Hashimoto thyroiditis, hypertension | RTX 1 gr in 2 injections 15 days apart. After 6 months, received a third RTX 1 gr injection | Not reported side effects | Clinical improvement with improved indexes of stiffness and spasm frequency | 100% | Anti-GAD ab serum antibodies  increased after treatment from 51 to 75 U/ml | Relapse after 6 months | Low number of patients.  Extreme variability of outcome measures and clinical assessment scales. |
| **Fekete**  **2012** | Case report | 1 | 12-year-old male patient; with 5-year disease history | RTX 500 mg/m^2^ body surface area given twice with an interval of 2 weeks | Not reported side effects | Improvement of gait. Reduction of frequency and severity of axial contractions, diminished startle response, and abolished sustained ankle clonus | 100% | Pre-treatment anti-GAD Ab was positive at 4,405 nmol/l.  Post-treatment anti-GAD Ab levels were not  available. | No information about the follow-up | Low number of patients.  Extreme variability of outcome measures and clinical assessment scales. Missing ab titers. |
| **Dalakas 2017** | Double-blind, placebo-controlled study | 12 | Age 50.8 years ± 8.4; disease history 8.0 years ± (4.3); Mean Baseline GAD titers (IU/ml) 1.003,000  Female (%) 6 (50.0)  Hyperthyroid (%) 3 (25.0)  Diabetes (%) 4 (33.3) | 24 patients (12 received RTX 1 gr bi-weekly vs placebo | No side effects | At 6 months the stiffness index was equally reduced in both groups. QOL scores improved significantly at 3 months in both groups, but not at 6 months | 100% | A marked reduction in the anti-GAD ab titers was observed in the RTX arm at month 6, but the effect was not statistically significant | At 6 months, improvement persisted in one patient in the placebo group vs  three out of 4 in the RTX group | Considerable placebo effect.  Extreme variability of outcome measures and clinical assessment scales. |
| **Kodama 2020** | Case-report | 1 | A 42-year- old male patient | RTX 375 mg/ m^2^ given four times with an interval of 1 week. | Not reported side effects | Prominent improvement of diplopia, involuntary upward drift, downbeat nystagmus, improved gait instability enabling him to walk straight without assistance | 100% | No change in anti-GAD ab titers (from 18,000 IU/mL to 17,000 IU/mL).  CSF manifested a slight decrease from 430 IU/mL to 340 IU/L. | We have no information regarding any relapses during the follow-up | Low number of patients.  Extreme variability of outcome measures and clinical assessment scales. Missing ab titers. |
| **Bai 2022** | Retrospective observational study | 4 | Fifty-five patients were included: 40 (72.73%) were women and initial neurological symptoms developed at 42(34-55) years of age. | 55 patients (4 patients received RTX) | Not reported side effects | Doubtful, not really predictable clinical improvement. 1 of 4 patients reported a clinical improvement | 25% (¼) | Not specified ab titers modification after RTX treatment | We have no information regarding any relapses during the follow-up | Retrospective study design. Extreme variability of outcome measures and clinical assessment scales. Missing ab titers and RTX dosage. |

Antibodies (Ab); Cerebrospinal fluid (CSF); Diabetes Mellitus (DM); Anti-Glutamic acid decarboxylase antibody (anti–GAD Ab); Patient/Problem, Intervention, Comparison, Outcome (PICO); Quality of life (QOL); Rituximab (RTX); Stiff-person syndrome (SPS); Thyroid-associated orbitopathy (TAO)
